# Assessing how emergency and trauma ultrasonography is taught to medical students

**DOI:** 10.31744/einstein_journal/2019AO4469

**Published:** 2019-02-01

**Authors:** José Cruvinel, Vinicius Rinaldi Vieira Marcondes, Marcelo Augusto Fontenelle Ribeiro

**Affiliations:** 1 Universidade de Santo Amaro, São Paulo, SP, Brazil.; 2 Hospital São Luiz, São Paulo, SP, Brazil.

**Keywords:** Ultrasonography, Multiple trauma, Emergency medicine, Teaching, Educational measurement, Ultrassonografia, Traumatismo múltiplo, Medicina de emergência, Ensino, Avaliação educacional

## Abstract

**Objective:**

To evaluate a method aimed at teaching ultrasound techniques to medical students in emergency settings.

**Methods:**

A prospective study conducted with 66 sixth-year undergraduate medical students. Students participated in theory and practicing sessions with a 5-hour load; knowledge acquisition was assessed through pre- and post-course and 90-day tests. A questionnaire were distributed to the students after course completion for theoretical and practical knowledge assessment.

**Results:**

Average pre-test grade in theoretical content evaluation was 4.9, compared to 7.6 right after course completion, and 5.9 within 90 days (p<0.001). Questions addressing technical aspects and image acquisition were mostly answered correctly; in contrast, questions related to clinical management of patients tended to be answered incorrectly. In practical evaluation, 54 students (81.8%) were able to correctly interpret images.

**Conclusion:**

Ultrasound applicability and image acquisition techniques can be taught to medical students in emergency settings. However, teaching should be focused on technical aspects rather than clinical management of patients.

## RESUMO

**Objetivo:**

Avaliar metodologia para o ensino de técnicas de ultrassom para estudantes de Medicina em ambiente de emergência.

**Métodos:**

Estudo prospectivo realizado com 66 alunos do sexto ano de graduação em Medicina. Os alunos participaram de aulas teóricas e práticas com carga de 5 horas, e os conhecimentos adquiridos foram avaliados em provas antes, logo depois e 90 dias após o curso. Após a conclusão das aulas, foi distribuído um questionário aos alunos, tendo sido avaliados os conhecimentos teórico e prático.

**Resultados:**

Nas avaliações teóricas, a média do grau de pré-teste foi de 4,9, com aumento observado para 7,6 após o término do curso, e de 5,9 para o teste de 90 dias (p<0,001). Questões sobre aspectos técnicos e aquisição de imagens foram mais frequentemente respondidas, e aquelas relacionadas ao manejo clínico foram as mais respondidas incorretamente. Na avaliação prática, 54 alunos (81,8%) conseguiram interpretar as imagens.

**Conclusão:**

Foi possível ensinar o uso de técnicas de ultrassom em um ambiente de emergência para estudantes na faculdade de Medicina e instruí-los em técnicas de aquisição de imagens, mas a instrução deve se concentrar em aspectos técnicos, e não em gerenciamento clínico.

## INTRODUCTION

History-taking and physical examination are critical aspects of semiology. However, some common adverse conditions in emergency and urgent care settings, such as high levels of noise, limited space between stretchers, inability to mobilize patients and patient clinical status, may prevent proper execution of these propedeutic techniques.

Classic semiology is also ineffective in 10% of high-energy trauma patients presenting with signs of abdominal injury during physical examination, or in cases of moderate to severe traumatic brain injury with non-specific physical signs, which may confuse the examining physician.^(^ ^1^ ^,^ ^2^ ^)^ The same applies to clinical emergencies involving hemodynamically unstable patients requiring monitoring, when reliable hemodynamic data cannot be obtained from vital signs, measurements of central venous pressure or even pulmonary artery catheter.^(^ ^3^ ^)^ Therefore, classical propedeutic procedures may fail to contribute relevant information required for decision-making in urgent and emergency care of trauma or non-trauma patients.

The advent of novel technologies has made it possible to develop bedside ultrasound techniques aimed to assess patients in these critical situations, in an effort to improve diagnosis and management. Examination of bedridden patients without having to transfer them to a different sector, immediate image analysis and the possibility to dispense without contrast agents and perform serial exams are some of the advantages of bedside ultrasonography (US). This technique can also be used for hemodynamic assessment, guided venous and arterial puncture, intracranial pressure measurement and deep vein thrombosis diagnosis.^(^ ^4^ ^,^ ^5^ ^)^

Teaching programs aimed at medical students or residents seeking bedside US training are currently scarce in Brazil. One of these programs is offered by *Faculdade Israelita de Ciências da Saúde* (FICS) and combines bedside US techniques with Team Base Learning (TBL) in the first undergraduate years. Another option would be training courses provided by several medical societies and hospitals, albeit with an average load of 8 hours, including theoretical and practicing sessions.^(^ ^6^ ^)^ Given the operator-dependent nature of US, courses must define different levels of training and limits, and establish the advantages of appropriate use of US by integrating physical examination and clinical data, so as to meet learning requirements and allow the acquisition of the necessary competence for routine application of the technique.^(^ ^7^ ^)^ Physicians need to develop cognitive and psychomotor skills before they can incorporate US into their clinical practice.^(^ ^8^ ^)^ The ability to interpret images precedes mastery of image acquisition techniques due to errors associated with poor gain adjustment and ensuing inappropriate image depth, which can be overcome with persistent training.^(^ ^9^ ^)^ Bedside US specialists are thought to lose the skill level required to acquire, interpret and understand images within approximately one year.^(^ ^10^ ^)^

The inclusion of this new technology in medical residency and even undergraduate programs is recommended to prevent lack of continuity and facilitate the learning curve.^(^ ^11^ ^-^ ^13^ ^)^ However, this remains to be accomplished in most services in Brazil. Alternatively, bedside US may be integrated into the medical education syllabus in an effort to mitigate ultrasound education *deficits* .

## OBJECTIVE

To evaluate the current model employed to teach urgent and emergency ultrasonography to undergraduate medical students.

## METHODS

A prospective study conducted at the *Hospital Geral do Grajaú* , at São Paulo (SP), Brazil, with sixth-year undergraduate medical students at *Universidade Santo Amaro* , from May to September 2015. This study was submitted to and approved by the Ethics Committee of the institution, protocol 1.070.632/20015, CAAE: 45243215.6.0000.5447. All undergraduate students agreed to participate and signed an informed consent. They were allocated to groups of eight or nine students according to medical school rotations, and were submitted to a workload of 5 hours, including theory and practicing sessions.

Program content distribution is shown in table 1 . Following an introductory theory session to each topic, acoustic windows were demonstrated and practiced by all students. The same young male mannequin was used for all groups; theory and practicing sessions were taught by the same professional, namely a certified expert in urgent and emergency US.

**Table 1:** Syllabus content

Description	Category	Hour load (minutes)
Basic aspects of ultrasonography and transducers	Theory session	40
FAST	Theory session	40
Pulmonary ultrasound	Theory session	40
Practical session	Practical session	40
Hemodynamic evaluation - cardiac windows and vena cava	Theory session	40
Guided vascular access	Theory session	20
Practical session	Practical session	40
Case discussion	Interactive session	40
Total		300

FAST: Focused Assessment with Sonography for Trauma.

Practical sessions began with demonstrations of Focused Assessment with Sonography for Trauma (FAST) and acoustic windows of the lung; students then practiced these until adequate visualization had been achieved. The following parameters were considered appropriate for these windows: hepatorenal space (visualization of the diaphragm, liver and kidneys); splenorenal space (visualization of the diaphragm, spleen and kidneys); perivesical space (visualization of the extra- and intraperitoneal portions of the bladder); pericardium (visualization of the heart and pericardium); lung (visualization of pleural sliding between two ribs).

Practical sessions continued with demonstrations of the long and short parasternal windows, apical window and vena cava. Visualization of the following structures was deemed appropriate in these cases: parasternal long window (left ventricle, mitral valve and left atrium); parasternal short window (left ventricle and papillary muscles); apical window (four cardiac chambers); and inferior vena cava (inferior vena cava at right atrium junction).

Students were evaluated for acquisition of theoretical and practical knowledge. Theoretical knowledge evaluation consisted of a ten-question multiple-choice test ( Appendix 1 ) completed prior to, right after and 90 days after the course (pre-course, post-course and 90-day tests, respectively). Test questions addressed technical aspects, FAST, the lung, hemodynamic assessment, vascular accesses and clinical applications of US. Practical evaluation addressed acquired skills, as follows: one out of five pre-determined skills (hepatorenal, splenorenal, perivesical, lung or cardiac acoustic window) was selected through a draw; students then had 5 minutes to demonstrate corresponding images using Venue 50^®^ ultrasound system (GE Healthcare, USA).

Students were then asked to complete a questionnaire addressing changes noted after the course, including the degree of subject knowledge enhancement. Questionnaire responses were rated according to the following Likert scale: 1 for nothing; 2, little; 3, neutral; 4, very much; and 5, extremely. Data analysis was based on average scores.^(^ ^14^ ^)^ Students averaging 4 or over were thought to have provided satisfactory answers.

Categorical variables (sex, evaluation, practical skills and specialty of choice) were described using frequency distributions; numerical variables (age, final grades and course time) were described using measures of central tendency and variability. Associations between final grades according to test application time (prior to, just after or 90 days after the course) were investigated using analysis of variance with repeated measures. Data normality (final grades) was tested using the Shapiro-Wilk test. Box-plots were used to display final grade data distribution. The level of significance was set at 5%. Statistical analyses were performed using software (STATA version 10.0).

## RESULTS

**Figure 1:**
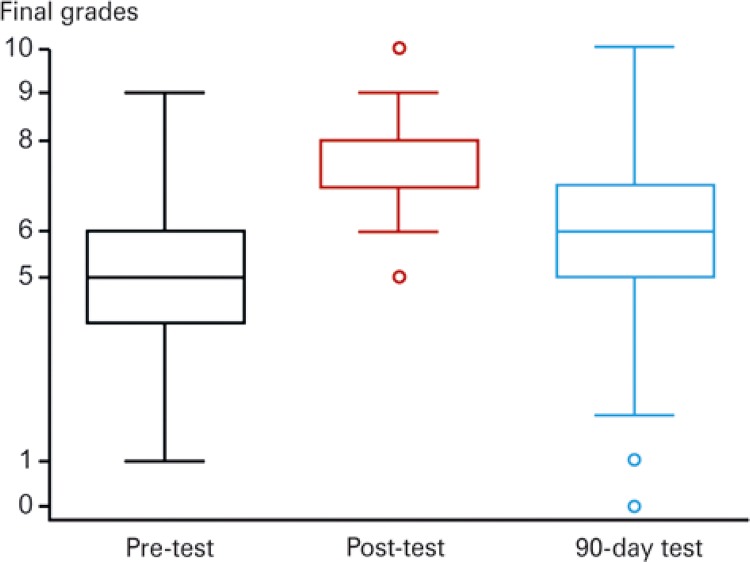
Test grade distribution per quartile. Black, red and blue plots represent pre-course, post-course and 90-day test grades, respectively

The mean age of 66 students was 25.8 years and most (65.2%) were women. Average pre-course grade (theoretical content evaluation) was 4.9, compared to 7.6 just after the course and 5.9 3 months later (p<0.001). Final grade distribution is shown in figure 1 . Only one student failed to complete the 90-day test.

Pre-course, post-course and 90-day test questions are listed in table 2 . Higher success rates were clearly associated with questions addressing technical aspects, FAST and hemodynamic assessment. Most students failed to provide correct answers to questions addressing clinical applications of US, even after course completion. Overall, the number of correct answers decreased between post-course and 90-day tests.

**Table 2:** Distribution of responses, by topics of questions

Topics of questions	Answers	Tests			
	
		Pre	Post	90-day	p value
		n (%)	n (%)	n (%)	
Technical aspects	Wrong	46 (69.7)	6 (9.1)	28 (43.1)	<0.001
	Correct	20 (30.3)	60 (90.9)	37 (56.9)	
Technical aspects	Wrong	43 (65.2)	7 (10.6)	19 (29.2)	<0.001
	Correct	23 (34.8)	59 (89.4)	46 (70.8)	
Clinical application	Wrong	37 (56.1)	55 (83.3)	50 (76.9)	<0.001
	Correct	29 (43.9)	11 (16.7)	15 (23.1)	
FAST	Wrong	36 (54.6)	2 (3.0)	25 (38.5)	<0.001
	Correct	30 (45.4)	64 (97.0)	40 (61.5)	
Clinical application	Wrong	48 (72.7)	60 (90.9)	54 (83.1)	0.018
	Correct	18 (27.3)	6 (9.1)	11 (16.9)	
Lung	Wrong	62 (93.9)	9 (13.6)	36 (55.4)	<0.001
	Correct	4 (6.1)	57 (86.4)	29 (44.6)	
Clinical application	Wrong	22 (33.3)	3 (4.6)	18 (27.7)	<0.001
	Correct	44 (66.7)	63 (95.4)	47 (72.3)	
Vascular access	Wrong	16 (24.2)	10 (15.2)	10 (15.4)	<0.001
	Correct	50 (75.8)	56 (84.8)	55 (84.6)	
Hemodynamic evaluation	Wrong	14 (21.2)	1 (1.5)	8 (12.3)	<0.001
	Correct	52 (78.8)	65 (98.5)	57 (87.7)	
FAST	Wrong	13 (19.7)	6 (9.1)	9 (13.8)	
	Correct	53 (80.3)	60 (90.9)	56 (86.2)	<0.001

p value obtained by Cochran’s Q test.

FAST: Focused Assessment with Sonography for Trauma.

As regards practical evaluation, Skill III was more frequently selected (16 cases; 24.2%), followed by Skill V (15 cases; 22.7%), Skills II and IV (12 cases; 18.2%), and Skill I (11 cases; 16.7%). Fifty-four students (81.8%) were able to obtain correct images after classes, with 100% success rate limited to the acoustic window of the lung. Distribution per selected skill is shown in table 3 .

**Table 3:** Distribution per selected skills (66 cases)

Practical skill	Concept	

	Yes	No
	n (%)	n (%)
Skill I – hepatorenal window	10 (90.9)	1 (9.1)
Skill II – splenorenal window	8 (66.7)	4 (33.3)
Skill III – perivesical window	13 (81.2)	3 (18.8)
Skill IV – pulmonary window	12 (100.0)	0
Skill V – parasternal long-axis window	11 (73.3)	4 (26.7)
Total	54 (81.8)	12 (18.2)

Questionnaire responses revealed that most of students considered the inclusion of ultrasound training into medical education to be important. However, most reported not feeling confident to operate a bedside ultrasound machine after such limited training ( Table 4 ).

**Table 4:** Results of evaluation questionnaire after class, considering weighted averages from the Likert scale

Question	Min-Max	Average
The training time was ideal for acquiring knowledge	2-5	4.06
I acquired basic and technical knowledge of ultrasound	3-5	4.42
I am able to operate an ultrasound machine for bedside evaluation	2-5	3.56
I feel confident in operating an ultrasound machine	2-5	3.33
The course improved my patients’ care	3-5	4.36
Ultrasound training should be part of the undergraduate syllabus	2-5	4.89
I consider a portable ultrasound machine useful in my daily life	1-5	4.52
The course met my expectations	3-5	4.74
The course provided knowledge that is important for my future professional training	3-5	4.68
The course served as motivation to make decisions about my future medical specialty		3.17

Likert scale: 1 − none/nothing; 2 − little; 3 − neutral; 4 − very; 5 − extremely.

## DISCUSSION

Bedside US can be used as a work-up tool to guide quick and effective management of emergency trauma and non-trauma patients. The technique was initially investigated and applied by emergency physicians in the 1980s, and has been enjoying rapid expansion over the last 20 years.^(^ ^15^ ^)^Bedside US is applicable to a myriad of medical conditions, as a procedural guiding or diagnostic assessment modality.^(^ ^16^ ^)^ Ferrada et al., reported US-based management changes in 96% of cases involving trauma patients aged over 65 years.^(^ ^17^ ^)^ Hemodynamic resuscitation can also be easily be performed in all cases under US guidance, as it allows determination of crystalloid or vasoactive drug needs in shock reversion.^(^ ^18^ ^-^ ^20^ ^)^

Studies have shown that US techniques are best taught to undergraduate students in emergency and trauma settings.^(^ ^21^ ^-^ ^24^ ^)^ The major challenge is to define the best way to offer emergency and trauma US training as part of the medical undergraduate program. In June 2014, the Association for Medical Ultrasound held a forum aimed to design a roadmap for integration of US into the medical education syllabus.^(^ ^25^ ^)^

In a recent systematic review, Mohammad et al., analyzed 52 articles on bedside US teaching methods and concluded that 2-day courses would be ideal – 1 day dedicated to theory and practicing sessions in healthy human models (four hours each) and 1 day to practice on animal models and simulators, case discussion and videos.^(^ ^6^ ^)^ However, according to Hempel et al., only 12% of information acquired is retained within 14 days of course completion; authors of that study suggested shorter theory sessions and additional daily practice after the course may improve retention rates.^(^ ^26^ ^)^ Likewise, this study revealed a 55% greater retention rate in post-course compared to pre-course tests, and a 22% drop within 90 days. Therefore, short courses do not seem to provide effective US training and should be aimed at overall update and skill enhancement.

Lewiss et al., believed optimal competence depends upon the development of image acquisition and interpretation skills, and understanding of associations between US images and therapeutic decision-making.^(^ ^27^ ^)^ The most common errors observed over the course of the learning period are related to gain settings and insufficient image depth, failure to recognize anatomical structures and misinterpretation of free fluids. However, these deficiencies tend to be corrected as more exams are performed and more experience gained.^(^ ^9^ ^)^ As regards clinical applicability of US, the training offered proved insufficient for specific knowledge acquisition, as related questions addressed multidisciplinary issues and involved clinical management of patients, which require not only mastery of image acquisition techniques and interpretation skills, but also more specific knowledge of urgent and emergency care. Hence, the incorporation of urgent and emergency US techniques into medical training may be a good strategy to promote adequate, safe and supervised learning. Given technical skills must be acquired along with other medical course disciplines and later during specialized medical training, US teaching should be aimed at technical skill acquisition rather than clinical management of patients.

Image acquisition ability assessment revealed that most of the students were able to obtain correct images, although 100% accuracy was limited to the acoustic window to the lung, as previously reported; the splenorenal window was associated with the highest level of difficulty (66.7% accuracy).^(^ ^28^ ^)^ Bedside US techniques were not associated with high levels of difficulty; therefore, images acquired were probably highly accurate.

Most students considered the teaching of urgent and emergency US in undergraduate medical education to be very important for improved patient care and knowledge acquisition for future training; however, they did not feel competent to operate an US machine in clinical practice.^(^ ^13^ ^,^ ^21^ ^,^ ^29^ ^,^ ^30^ ^)^

As regards US teaching methodology in this study, some limitations should be taken into account. First, students were taught and evaluated by the same professional. Second, the level of difficulty of questions interrogating theoretical content required deeper specific knowledge of clinical behaviors beyond student capabilities at that point in time. Short course duration is yet another limitation. Also, the fact that undergraduates had not received any ultrasound training in preclinical years may have negatively impacted their performance in proposed assessments, as they lacked the necessary clinical background. Further studies should be conducted at different institutions for comparative analysis of teaching methods and performance in assessments aimed exclusively at US imaging skills acquired over the course of undergraduate training.

## CONCLUSION

The teaching of urgent and emergency ultrasonography can be incorporated into the medical education syllabus and should enable students to learn how to acquire ultrasound images and promote further clinical reasoning development.

## Appendix

**Table:**

Appendix 1 – QUESTIONS			
1. As regards the technical aspects of ultrasound, which of the following is incorrect?			
a) The M-mode is the motion mode.			
b) The B mode is the most widely used.			
c) The ultrasound effect is achieved by pulsing a crystal and sending energy waves to the patient.			
d) In Doppler US, arteries are depicted in red.			
2. As regards transducers:			
a) High frequency transducers are ideal for abdominal cavity assessment.			
b) The higher the frequency, the lower the tissue-penetrating capability.			
c) Low frequency transducers are better suited for venipuncture, as the procedure requires low depth penetration.			
d) Transducers consist of zinc and copper crystals.			
3. A patient was admitted after having been hit by a car. Physical examination revealed clear airways, normal breathing sounds, BP 120/80mmHg, HR 86bpm, painful abdomen with no signs of peritonitis and Glasgow 15. The best approach is to:			
a) Perform FAST.			
b) Recommend exploratory laparotomy.			
c) Perform diagnostic peritoneal lavage.			
d) Request abdominal CT.			
4. As regards FAST:			
a) The procedure should only be carried out on hemodynamically unstable patients.			
b) The perivesical window is the site of greater fluid accumulation.			
c) The procedure can be used to define hepatic and splenic injuries.			
d) The procedure allows detection of free abdominal fluid from 250mL.			
5. A patient with a history of fall presents with a penetrating lesion at the level of the 7^th^ left intercostal space. Ultrasound examination reveals lung sliding and negative FAST. What is the next course of action?			
a) Perform laparoscopy.			
b) Drain the chest at the level of the fifth intercostal space.			
c) Request contrast-enhanced abdominal CT.			
d) Stich the wound and send the patient home with appropriate prescriptions.			
6. Choose the correct alternative:			
a) Detection of lung sliding sign is consistent with pneumothorax.			
b) A-lines are consistent with pulmonary edema.			
c) Ultrasound is less sensitive and specific as compared to radiography.			
d) The lung point is useful in determining the size of the pneumothorax.			
7. Patient admitted to ICU with pulmonary source of infection, BP 80/40mmHg, HR 134bpm and receiving noradrenaline 0.8mcg/kg/minute. Bedside US revealed collapsed vena cava, good cardiac contractility and presence of A-lines on lung surface. What is the next course of action?			
a) Increase noradrenaline dose.			
b) Start dobutamine.			
c) Perform crystalloid fluid resuscitation.			
d) Add vasopressin.			
8. Ultrasound-guided vascular puncture:			
a) Is ideal for peripherally inserted central venous catheter.			
b) Is not applicable to arterial puncture aimed at invasive arterial pressure monitoring.			
c) Is limited to the internal jugular vein.			
d) Has no benefit over conventional punctures.			
9. Changes in inferior vena cava diameter:			
a) Are useful to assess hypovolemia.			
b) Cannot be used in peritoneal dialysis patients.			
c) Are reliable in patients under mechanical ventilation with high PEEP.			
d) None of the above.			
10. In which of the following situations would FAST be more sensitive to assess hemoperitoneum?			
a) Hypotensive patient sustaining blunt abdominal trauma.			
b) Stab injury patient with evisceration.			
c) Hemodynamically stable patient with open book pelvic fracture.			
d) None of the above.			
**ANSWERS**			
1	D	6	D
2	B	7	C
3	D	8	A
4	D	9	A
5	A	10	A
